# Postpartum Ogilvie's Syndrome After a Vaginal Delivery

**DOI:** 10.7759/cureus.58483

**Published:** 2024-04-17

**Authors:** Kelcie Lushefski, Christian H Summa, Camden Zemp, Timothy Farrell

**Affiliations:** 1 General Surgery, Geisinger Wyoming Valley Medical Center, Wilkes-Barre, USA; 2 General Surgery, Geisinger Community Medical Center, Scranton, USA

**Keywords:** abdominal distention, neostigmine, vaginal delivery, postpartum, ogilvie's syndrome

## Abstract

Ogilvie's syndrome is a colonic pseudo-obstruction that results in colonic dilation without a mechanical obstruction. We discuss a 33-year-old, 36-week pregnant, G1P0L0A0 female who presented with severe pre-eclampsia. Less than 24 hours after induction by vaginal delivery, she developed significant abdominal pain and distention. On a CT scan of the abdomen and pelvis, she was diagnosed with Ogilvie’s syndrome due to a finding of large bowel dilation with an abrupt transition point at the splenic flexure without a noted mass. She was initially treated conservatively with nasogastric tube decompression and IV fluid resuscitation. When these conservative measures failed, neostigmine was administered with transient improvement in symptoms. Despite the appropriate administration of neostigmine and initial relief of symptoms with stool output, the patient ultimately required surgical intervention with the creation of a transverse loop colostomy. The development of Ogilvie's syndrome in the postpartum period is a very rare finding, particularly after a vaginal delivery.

## Introduction

Ogilvie’s syndrome is a colonic pseudo-obstruction that results in colonic dilation without a mechanical obstruction. This syndrome generally manifests in frail, elderly individuals who reside in a nursing home, but may also be found in younger, healthy individuals in certain circumstances. These circumstances include pre-disposing conditions, such as trauma, infection, cardiac disease, post-Cesarian-section (C-section), and post-hip surgery [1]. Postpartum Ogilvie’s syndrome has been documented in case studies, but is generally associated with a C-section and is a rare occurrence following a vaginal delivery. A systematic review of 125 cases of postpartum Ogilvie’s syndrome found that 92% occurred after a C-section with only 8% occurring after a vaginal delivery [2]. Although no risk factors were able to be identified in these patients, the indications for C-section included pre-eclampsia, like in our patient, multiple pregnancies, and antepartum hemorrhage. In this subset of patients, 50% were successfully treated non-operatively with bowel rest, IV fluid resuscitation, and electrolyte repletion [2]. Not only is postpartum Ogilvie’s syndrome after vaginal delivery rare, but it is even more unusual to require surgical intervention. We describe a case of Ogilvie’s syndrome occurring after a vaginal delivery.

## Case presentation

We present a 33-year-old, 36-week pregnant, G1P0L0A0 female who presented to emergency department (ED) triage with epigastric abdominal pain, nausea, emesis, and constipation. She denied any vaginal bleeding, but she did notice decreased fetal movement over the prior three days. On physical exam, she had no abdominal or uterine tenderness, and had contractions every 4-6 minutes. On fetal monitoring, there was moderate variability without accelerations or decelerations. On cervical exam, she had 1 cm dilation, 50% effacement, -1 station, medium consistency, and in midposition. The patient was hypertensive with a BP of 150/81 and her urine protein/serum creatinine ratio was elevated at 321mg/g (reference <150mg/g). She was admitted to the obstetrics unit with a concern for pre-eclampsia with severe features. The patient continued to complain of 10/10 abdominal pain, now in the right upper quadrant, and an ultrasound (U/S) showed significant findings. Due to persistent symptoms with the concern for severe pre-eclampsia, a discussion was had about a Cesarean section; however, the patient was adamant that she only wanted to have a vaginal delivery. The decision was made to attempt induction through vaginal delivery; however, if this failed, she would then proceed to a C-section. Understanding the risks, she was induced that night and had a vaginal delivery, complicated by a first-degree perineal laceration, which was repaired using 3-0 Vicryl. 

**Figure 1:**
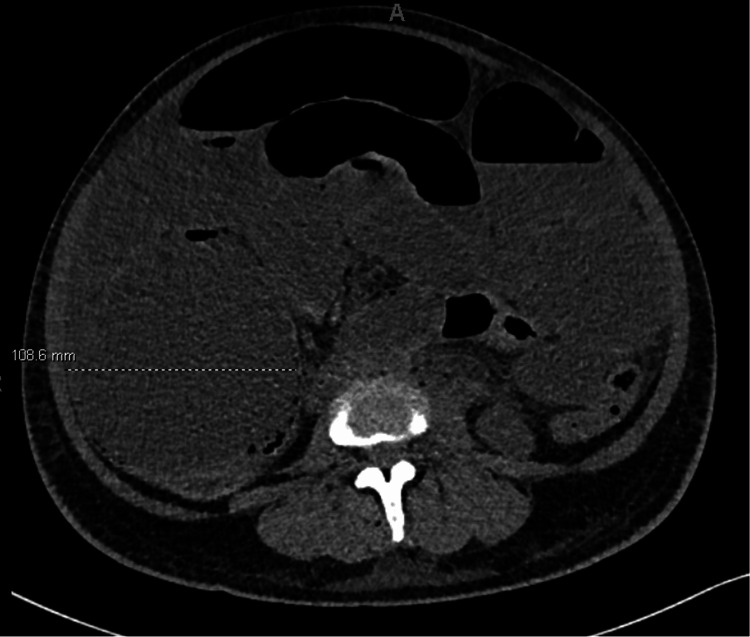
Coronal views of CT abdomen/pelvis showing a dilated cecum to 10.9 cm diameter

**Figure 2:**
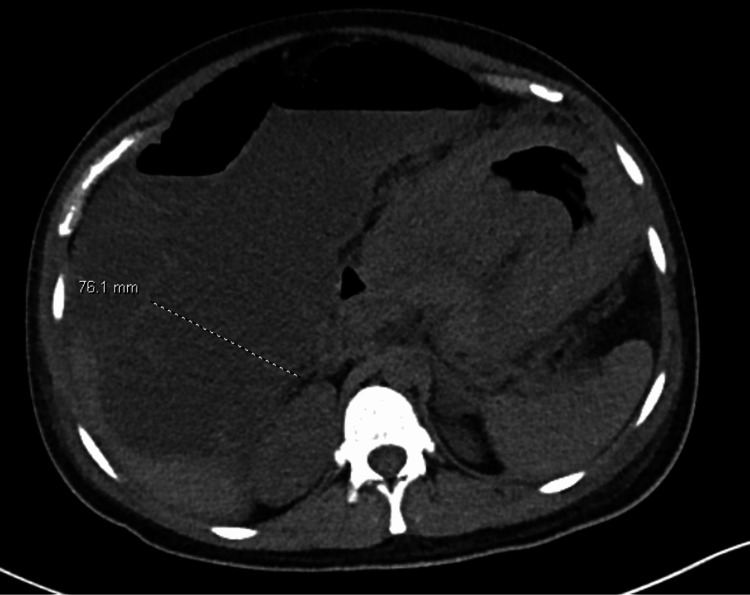
Coronal views of CT abdomen/pelvis showing ascending colon dilated to 7.6 cm diameter

Approximately 24 hours after delivery, the patient reported 10/10 abdominal pain, significant bloating, and nausea. On physical exam, she was noted to be markedly distended with diffuse tenderness to palpation. Labs were drawn, including a complete blood count (CBC) and comprehensive metabolic panel (CMP), noting hyponatremia of 130 mEq/L (normal range: 135-145 mEq/L), hypokalemia of 3.4 mEq/L (normal range: 3.5 to 5.2 mEq/L), and hypocalcemia of 6.6 mg/dL (normal range: 8.5 to 10.5 mg/dL), all of which were repleted. A CT abdomen/pelvis without contrast showed marked distention of both the small and large bowel with the cecum measuring 10.9 cm and ascending colon measuring 7.5 cm in diameter, with an abrupt transition from distended bowel to decompressed bowel at the splenic flexure. These findings were concerning for Ogilvie’s syndrome (Figures 1, 2). At this time, general surgery was consulted. The patient was initially managed conservatively with nasogastric (NG) tube decompression and IV fluid resuscitation while avoiding opioids and correcting electrolyte abnormalities. Approximately 1.5 hours later, she was re-examined. She was still having significant abdominal pain and distention, and her NG tube output was only 200 cc. At this time, it was decided to try neostigmine. 

The patient was taken to the pre-operative area with the plan to administer neostigmine there to allow continuous monitoring during and after administration. If this failed, the plan was to proceed directly to the operating room for either endoscopic decompression or surgical intervention, depending on the severity of the symptoms. Following administration of 5 mg of neostigmine intravenously, the patient reported initial improvement of symptoms with passage of a small amount of stool, flatus, and reduced abdominal pain. However, as her symptoms returned 45 minutes later with peritonitis, she was transported to the operating room for surgical intervention, where she underwent a transverse loop colostomy. During the operation, she had an immediate output of three liters from her colostomy, with an additional output of four liters over the next 24 hours. The patient felt significantly better post-operatively with decreased abdominal distention and tenderness. Her symptoms continued to improve, and she had an uncomplicated post-operative course. She was eventually discharged on post-operative day 5. 

## Discussion

Ogilvie’s syndrome is a relatively rare finding in a young, healthy patient. Although infrequent, there is an association with Ogilvie’s syndrome following a C-section, but it is quite rare following vaginal delivery. Despite this being a rare finding, it is very important to maintain a broad differential and recognize these symptoms in postpartum patients to appropriately manage their care. If not properly diagnosed, it can result in peritonitis, colonic perforation, and even death. Although this disease process is primarily found in elderly, frail individuals, the most common causes in young, healthy women include after a C-section, trauma, and pelvic surgery [3]. 

Although the pathophysiology of postpartum Ogilvie’s syndrome has not been well defined, it has been speculated that it may be caused by decreased parasympathetic colonic innervation, resulting in distal colonic atony and proximal colonic dilation [1]. This may appear as a transition point, or pseudo-obstruction, on imaging, but there is no true mechanical obstruction. In our patient, this appeared as a transition point at the splenic flexure. In postpartum patients, the pathophysiology is believed to be related to autonomic imbalance secondary to sacral parasympathetic nerve damage. These nerves innervate the colon and can become damaged during delivery due to their proximity to the vagina, cervix, and broad ligament [4]. 

Ogilvie’s Syndrome in the postpartum period is treated the same way as in any other patient population. It is important to first obtain imaging once it is suspected and to know that a cecal diameter of greater than 12 cm has a high risk of impending rupture. In our patient, as the cecum measured 10 cm, conservative management was initially attempted with NG tube decompression, IV fluid resuscitation, avoidance of opioids, and electrolyte repletion. When NG tube decompression and IV fluid resuscitation were not adequate, neostigmine was administered. 

Neostigmine is an acetylcholinesterase inhibitor that has been shown to have benefit in Ogilvie’s syndrome by improving colonic peristalsis [5]. A meta-analysis of 127 patients who were administered neostigmine for Ogilvie’s syndrome found that 89.2% of patients had complete resolution of symptoms after only 1 dose. Furthermore, this medication has a quick onset of less than 20 minutes and a short duration of action of less than 2 hours [6]. It is generally given as an intravenous bolus dose of between 2 and 5 mg. We administered 5 mg to our patient. Additional bolus doses can be attempted, but if this is unsuccessful, surgical intervention may be required. 

Surgery may become necessary for Ogilvie’s syndrome in patients who fail conservative management or those who have peritonitis or perforation. Patients who have peritonitis on physical exam or have imaging findings concerning for bowel perforation should promptly be taken to the operating room for an exploratory laparotomy, bowel resection for ischemia, and ostomy creation. Those without peritonitis or perforation but failing conservative management, like in our case, may also require surgical intervention. In these situations, a diverting loop colostomy may be performed to allow for decompression. Our patient underwent a transverse loop colostomy. This was chosen due to ease of eventual reversal and to allow decompression of the colon. Endoscopy has also been used in the treatment of Ogilvie’s syndrome when conservative measures, including neostigmine, fail. However, endoscopic intervention has a fairly high recurrence rate of 40% [7]. Due to the severity of symptoms in our patient (peritonitis), endoscopic decompression prior to operative intervention was not attempted. 

## Conclusions

In conclusion, Ogilvie’s syndrome is a rare finding in post-partum women, especially in those who had a vaginal delivery rather than a C-section. This syndrome must remain on the differential diagnosis in postpartum women with abdominal pain, abdominal distention, nausea, and vomiting. Surgical intervention with an ostomy creation may be required in individuals who are refractory to conservative management.
